# Blood Biomarkers for Monitoring and Prognosis of Large Vessel Vasculitides

**DOI:** 10.1007/s11926-021-00980-5

**Published:** 2021-02-10

**Authors:** Enrico Tombetti, Elvis Hysa, Justin C. Mason, Marco A. Cimmino, Dario Camellino

**Affiliations:** 1 Internal Medicine, Department of Biomedical and Clinical Sciences “Luigi Sacco”, Milan, Italy; 2 Internal Medicine and Rheumatology, Sacco and Fatebenefratelli Hospitals, Milan, Italy; 3 Research Laboratory and Academic Division of Clinical Rheumatology, Department of Internal Medicine, University of Genoa, Genoa, Italy; 4 National Heart and Lung Institute, Imperial College London, London, UK; 5 Rheumatology, Hammersmith Hospital, Imperial College NHS Trust, London, UK; 6 Division of Rheumatology, Musculoskeletal System Department, La Colletta Hospital, Local Health Trust 3 Genoa, Via del Giappone 3, 16011 Arenzano, Italy; 7 Autoimmunology Laboratory, Department of Internal Medicine, University of Genoa, Genoa, Italy

**Keywords:** Large vessel vasculitis, Giant cell arteritis, Takayasu arteritis, Serum biomarkers, Calprotectin, Pentraxin-3

## Abstract

**Purpose of Review:**

Large vessel vasculitides (LVVs) are inflammatory conditions of the wall of large-sized arteries, mainly represented by giant cell arteritis (GCA) and Takayasu arteritis (TA). The inflammatory process within the vessel wall can lead to serious consequences such as development of aneurysms, strokes and blindness; therefore, early diagnosis and follow-up of LVV are fundamental. However, the arterial wall is poorly accessible and blood biomarkers are intended to help physicians not only in disease diagnosis but also in monitoring and defining the prognosis of these conditions, thus assisting therapeutic decisions and favouring personalised management. The field is the object of intense research as the identification of reliable biomarkers is likely to shed light on the mechanisms of disease progression and arterial remodelling. In this review, we will discuss the role of blood biomarkers in LVVs in the light of the latest evidence.

**Recent Findings:**

In clinical practice, the most widely performed laboratory investigations are the erythrocyte sedimentation rate (ESR) and C-reactive protein (CRP). However, these indices may be within normal limits during disease relapse and they are not reliable in patients receiving interleukin-6 (IL-6) receptor inhibitors. New biomarkers struggle to gain traction in clinical practice and no molecule with good accuracy has been identified to date. IL-6, a pro-inflammatory cytokine that drives CRP synthesis and increases the ESR, is one of the most promising biomarkers in the field. IL-6 analysis is increasingly performed, and serum levels are more sensitive than ESR for active GCA and might reflect persistent inflammation with high risk of relapse in patients on IL-6 receptor inhibitors. A future with biomarkers that reflect different disease features is an important aspiration. Accordingly, intense effort is being made to identify IL-6-independent inflammatory biomarkers, such as S100 proteins, pentraxin-3 and osteopontin. Moreover, metalloproteinases such as MMP2/9 and angiogenic modulators such as VEGF, YLK-40 and angiopoietins are being studied as markers of arterial remodelling. Lastly, biomarkers indicating organ damage may guide prognostic stratification as well as emergency therapeutic decisions: the most promising biomarkers so far identified are NT-proBNP, which reflects myocardial strain; pentraxin-3, which has been associated with recent optic nerve ischemia; and endothelin-1, which is associated with ischaemic complications.

**Summary:**

Currently, the use of these molecules in clinical practice is limited because of their restricted availability, lack of sufficient studies supporting their validity and associated costs. Further evidence is required to better interpret their biological and clinical value.

## Introduction

Large vessel vasculitides (LVVs) are characterised by idiopathic inflammation involving the large-sized arteries such as the aorta, the pulmonary artery and their main branches [1]. The prototypic LVV is Takayasu arteritis (TA), a rare condition, typically affecting young women [2], characterised by chronic granulomatous arterial inflammation causing arterial wall thickening and potentially resulting in luminal remodelling with arterial steno-occlusions or, more rarely, dilatation or aneurysms. Although the inflammatory nature of TA is well recognised, the mechanism of arterial remodelling and its pathogenic link with inflammation are poorly understood [3]. Clinical features relate to a systemic inflammatory response of variable intensity, to cardiovascular complications including arterial hypertension and to direct consequences of arterial involvement such as end-organ ischemia, vascular bruits, aortic regurgitation or aneurysm rupture. The course of TA is characterised by multiple disease flares in 80% of patients, although the vast majority eventually undergo a spontaneous remission characterised by residual arterial damage [4].

Giant cell arteritis (GCA) is the other major variant of LVV [1], representing the most common vasculitis in the elderly [5••]. GCA is characterised by inflammation in the large- and medium-sized arteries with two different clinical patterns, which frequently overlap. The first, often described as the predominant ‘cranial-GCA’, is characterised by the involvement of the extra-cranial branches of the carotid artery including the temporal artery [6]. The associated clinical manifestations are new-onset headache, scalp tenderness, thickening of superficial temporal arteries and anterior ischemic optic neuropathy (AION), which can rapidly lead to irreversible blindness if glucocorticoid (GC) treatment is delayed or its dosing is insufficient. The term ‘temporal arteritis’, often used when referring to GCA, derives from this pattern of presentation. Predominant large vessel (LV)–GCA is at the other end of the GCA spectrum and characterised by a robust systemic inflammatory response, aortitis and limb claudication [7••]. The long-term consequences of LV-GCA may be vascular stenoses and aneurysms. Polymyalgia rheumatica (PMR) is characterised by inflammatory pain and stiffness in the shoulder and pelvic girdles, often associated with constitutional symptoms [8]. Up to 50% of GCA patients presenting with polymyalgic features and 10–30% of patients with PMR show LVV on imaging or histological features of GCA at temporal artery biopsy (TAB) [9]. A typical feature of GCA is an intense systemic inflammatory response and the majority of GCA and PMR patients display increased inflammatory markers [10, 11].

Arteries represent immune-privileged sites endowed with intrinsic tolerogenic properties and devoid of resident leukocytes, with the notable exception of vascular dendritic cells [12, 13]. Pathogenesis of LVV is believed to represent the consequence of an immune response, mainly represented by cell-mediated immunity, toward an unknown antigen within the arterial wall. Inflammatory cells including macrophages and lymphocytes gain access to the arterial wall via the vasa vasorum following vascular dendritic cell activation, release of chemoattractants and expression of adhesion molecules by vasa vasorum endothelium. In turn, recruitment of inflammatory cells predisposes to direct tissue injury and further enhances the production of mediators of inflammation and tissue remodelling, such as growth factors and matrix metalloproteinases (MMPs) [12, 13].

No specific biomarker is available for the diagnosis or the follow-up of LVV. Accordingly, the diagnosis is usually made on the basis of the clinical presentation and supported by the use of imaging or histology. Patient follow-up is based upon physical examination, laboratory investigations and imaging techniques [14–16].

In this review, serological biomarkers for diagnosis, prognosis and monitoring of LVVs are discussed in the light of the most recent literature.

## Serological Biomarkers for Diagnosis of LVV

Despite the absence of specific biomarkers for diagnosing LVVs, several serological or cell-associated molecules have been evaluated, aiming to reduce the diagnostic delay frequently encountered in these conditions. A selection of biomarkers supporting the diagnosis of TA and GCA is provided in Table 1.

**Table 1:** A selection of blood biomarkers with their role in different disease phases in patients with TAK and GCA

Biomarker	Utility for diagnosis	Utility for prognosis	Utility for monitoring and follow-up	Availability	Advantages	Disadvantages
ESR [4, 10, 11, 24, 77]	++	+	++	+++	Widely available and inexpensive. Greater utility for diagnosis and prognosis in GCA than in TA.	Influenced by concomitant conditions (e.g. age, anaemia). Not specific for vasculitis. Inaccurate to detect arterial remodelling. Unreliable during anti-IL6 treatments.
CRP [4, 10, 11, 24, 57, 77]	++	+	++	+++	Widely available and inexpensive. More specific than ESR. Greater utility for diagnosis and prognosis in GCA than in TA.	Not specific for vasculitis. Inaccurate to detect arterial remodelling. Unreliable during anti-IL-6 treatments.
IL-6 [33, 35, 36, 85]	+	–	+	++	Possible role in predicting relapse and detecting infections in patients treated with TCZ.	Lower availability compared with traditional biomarkers. Not defined diagnostic value in GCA.
SAA [30, 32, 80, 81]	Not defined	+	+	++	Potential biomarker of arterial inflammation.	Not specific for arteritis. Increased in different conditions, including bacterial infections. Lower availability compared with other biomarkers
PTX-3 [38, 39, 87, 111]	+	+	+	+	Potential biomarker of subclinical vascular inflammation. In GCA, possible role in detecting recent optic nerve ischemia	Lower availability compared with other biomarkers.
NT-proBNP* [58]	–	+++	+	+++	Biomarker of myocardial strain with prognostic value.	Concentrations rise in cardiac and renal failure, independent of TA.
MMP-2/9* [100]	Not defined	Not defined	+	+	Potential biomarker of arterial remodelling.	Lower availability compared with other biomarkers.
OPN^#^ [71]	Not defined	+	+	+	Possible role in predicting relapse and monitoring disease activity in patients receiving anti-IL-6 pathway treatments.	Lower availability compared with other biomarkers.
S100 proteins^#^ [72, 104]	+	+	+	++	Possible role in monitoring disease activity, less influenced by anti-IL-6 pathway treatments than ESR and CRP.	Lower availability compared with other biomarkers.

*Studies focused on TA. ^#^Studies focused on GCA. *CRP* C-reactive protein, *ESR* erythrocyte sedimentation rate, *GCA* giant cell arteritis, *IL-6* interleukin-6, *MMP-2/9* matrix metalloproteinase 2 and 9, *NT-proBNP* aminoterminal pro-B-type natriuretic peptide, *OPN* osteopontin, *PTX-3* pentraxin-3, *SAA* serum amyloid A, *TAK* Takayasu arteritis, *TCZ* tocilizumab

### Acute Phase Reactants Included in Routine Laboratory Investigations

Acute phase reactants performed among routine laboratory investigations include erythrocyte sedimentation rate (ESR) and C-reactive protein (CRP). These examinations are inexpensive and widely used in clinical practice. CRP is directly produced by the liver in response to interleukin (IL)-6 [17, 18]. Conversely, a raised ESR is related to multifactorial mechanisms, including increased levels of fibrinogen, alpha-1 and gamma-globulins, as well as reduced plasma albumin and anaemia [19, 20]. ESR increases progressively with age, and this is believed to reflect the accrual of oxidative damage and the progressive establishment of a pro-inflammatory milieu in the elderly, collectively known as ‘inflammaging’ [21]. There is no consensus concerning the upper limits of normal for the ESR, although a simple rule identifies the cut-off at age/2 (+ 5, if female) [22]. However, no cause was identified in up to 47% of subjects with an ESR between 50 and 99 mm/h [23], limiting the specificity of such marker.

Elevation in the concentration of acute phase reactants is frequent during active TA and may help to uncover an inflammatory state and pave the way for further workup. The intensity of the systemic inflammatory response is highly variable and normal acute phase reactants were observed in 10–30% of patients in some studies [4, 24••]. A proportion of these patients [4, 24••], however, were diagnosed in an inactive phase of TA and it is unclear whether they might have experienced a prior systemic inflammatory response.

In GCA, a highly intense systemic inflammatory response is typical. Indeed, ESR values above 50 mm/h are part of the 1990 classification criteria of the American College of Rheumatology (ACR) for GCA [25]*.* However, in a cohort of 177 patients with biopsy-proven GCA [10], eighteen (10.2%) showed normal values of both ESR and CRP (defined as an ESR value of ≤ 22 mm/h in men and ≤ 29 mm/h in women, and a value of CRP ≤ 8 mg/L), at the time of diagnosis. It should be noted that only 7 of these 18 patients were GC-naïve at the time of the biopsy and this might have influenced the results. Visual disturbance despite normal ESR and CRP was present in 3 of the 7 patients, of whom one had visual loss. A systematic review showed the percentage of patients with GCA with normal inflammatory indices may range from 0 to 22% [26].

Another major acute phase parameter is serum amyloid A (SAA), synthetised and secreted by the liver in different isoforms and at inflamed sites including the temporal arteries [27]. It has been recently observed that this SAA participates in blood vessel modification, invasion and destruction [28]. SAA also induces the synthesis of IL-6 and has proangiogenic properties through upregulation of vascular endothelial growth factor receptor-2 (VEGFR-2) [29]. A cross-sectional study on a cohort of 99 TA patients (with a mean disease duration of 73 months) showed higher levels of serum SAA as compared to healthy controls [30]. Despite mean SAA levels significantly differed from TA subjects and controls, overlap was substantial and patients could not be accurately discriminated by using SAA serum levels alone [30].

In GCA, SAA serum concentration levels significantly differ between untreated GCA and healthy controls, with an average 83-fold increase in the former as compared to controls [31]. Interestingly, SAA concentrations were the strongest positive correlate of IL-6 levels [31]. No study has compared SAA in treatment-naïve GCA and treated active or inactive disease versus healthy controls. Being identified as a marker of disease activity, SAA may also be helpful in defining a relapse [32]. However, similar to ESR and CRP, as SAA levels may increase also in other conditions (such as microbial infections), its value for diagnosing GCA appears to be limited.

### Other Potential Biomarkers

Considering the inflammatory milieu of LVV, biomarker research in the field has studied several other inflammatory molecules. IL-6 is one of the pivotal pro-inflammatory cytokines and may be locally generated in the inflamed arteries both in TA and GCA [33, 34]. High IL-6 release is considered to account for the prominent systemic inflammatory symptoms observed in several patients [12]. Despite its key role in LVV pathogenesis and the efficacy of IL-6-targeted therapies, no study has verified the diagnostic value of serum IL-6 for GCA and TA. Indeed, although IL-6 measurement is becoming increasingly available in many laboratories, it is likely to have similar limitations to the analysis of ESR and CRP despite much higher costs. Considering the insufficient evidence to support its use, IL-6 analysis is seldom requested in current clinical practice. Serum IL-18 is raised both in TA and GCA [12, 35–37]. IL-18 is a pro-inflammatory cytokine believed to be produced predominantly by vascular antigen-presenting cells which activate Th1 and Th17 cells [12]. However, an important overlap in IL-18 levels was observed between LVV patients and controls, with poor discrimination of LVVs from other inflammatory conditions [12, 35–37].

Pentraxin-3 (PTX-3) is an acute phase protein synthetised locally at sites of inflammation, where it is believed to exert important actions in the opsonisation of self and foreign antigens and to contribute to structural and functional activity of the extracellular matrix. Plasma levels of PTX-3 were elevated in TA and GCA [38, 39], as well as in other vascular conditions such as atherosclerosis, acute myocardial infarction and following acute vascular damage during stenting procedures [40–43].

Considering that the pathogenesis of LVV involves multiple branches of the immune system (antigen-presenting cells, T and B cells), research has focused on autoantibodies. However, specific autoantibodies have never been reported in the LVVs and the role of B cells in these conditions remains elusive. However, several autoantibodies have been identified, including anti-endothelial (AECA), antiphospholipid and anti-ferritin antibodies. A specific pathogenic role for these autoantibodies remains to be demonstrated, since all conditions characterised by tissue injury may elicit autoantibody production. AECA are widely reported in the vasculitides including LVV. Although AECA have been associated with LVV disease pathogenesis, their precise role in disease development and progression is not defined. Antigenicity remains of interest and recent studies have identified endothelial protein C receptor and scavenger receptor class B type 1 as AECA antigenic targets in TA [44].

Antiphospholipid antibodies have been observed in 30–80% of patients with GCA [45–48] and in up to 41% with TA [49]. The potential use of these antibodies as activity markers is discussed below. Anti-ferritin antibodies have been identified in 92% of untreated active GCA patients (with progressively lower prevalence in patients with flares during therapy and in inactive GCA) [50], and also in 62% of TA patients [51]. These antibodies were found to be less abundant in other diseases and healthy controls. Further studies are required to confirm these results and to define any potential pathogenic role and clinical utility [52].

An alternative strategy has been adopted in studies evaluating multiple potential biomarkers in parallel. In TA, screening based on a 440-cytokine protein array [53] identified higher plasma levels of tissue inhibitor of metalloproteinases (TIMP)-1, P-cadherin and MMP-9 and lower levels of WNT inhibitory factor (WIF)-1 in the plasma of TA patients as compared to healthy controls. A validation in two independent cohorts confirmed higher plasma levels of TIMP-1 and P-cadherin as compared to controls. P-cadherin is a cell adhesion molecule involved in multiple processes including cell migration and growth, while TIMP-1 is a metalloproteinase inhibitor with a proposed role in fibrotic conditions and it may be speculated that this protein contributes to vascular remodelling and arterial wall fibrosis. The involvement of metalloproteinases in vascular remodelling is also highlighted by findings from another study that found higher levels of MMP-9 in peripheral blood in GCA [54]. Increased MMP-9 mRNA was detected in the lamina media of the inflamed arteries, a finding consistent with the pathogenic model of local synthesis of matrix-remodelling factors in LVVs.

Recent studies have evaluated blood biomarkers that might help to identify occult GCA in the setting of PMR, by comparing patients with or without LV-GCA in FDG-PET. One study found that ESR and the concentrations of both the soluble tyrosine kinase receptor Tie-2 and angiopoietin-2, markers of angiogenesis, were significantly elevated in patients with PMR and LV-GCA as compared to those with PMR only [55••]. Angiopoietin-2 outperformed the discriminative potential of both CRP and ESR. Another study indicated that low levels of MMP-3 might indicate the presence of underlying GCA in patients with clinical PMR [56].

## Serological Biomarkers for Disease Phenotype Assessment and Prognosis

Few data are available on blood biomarkers for assessment of GCA or TA phenotypes (such as distinction of cranial-GCA from LV-GCA) and disease prognosis. In this section, the role of blood biomarkers in defining disease phenotypes will be discussed along with their prognostic value at time of diagnosis.

In TA, elevated concentrations of CRP using a high-sensitivity assay predicted the occurrence of major cardiac events [57]. Moreover, NT-proBNP may identify patients with cardiovascular complications and heart failure, and higher levels of NT-proBNP were reported in patients with severe TA according to Ishikawa’s criteria [58]. In GCA, an intense acute phase response is associated with a higher risk of relapse [59]. In fact, it has been proposed that this might reflect either insufficient treatment or a more resistant disease [60]. Interestingly, multiple papers report that high concentrations of acute phase proteins at baseline might predict GCA relapses [61, 62] and inflammatory anaemia has been reported to predict disease flares [63].

In GCA, one of the most feared complications is optic nerve ischemia, which occurs almost exclusively before initiation of glucocorticoid therapy [64]. Gonzalez-Gay et al. compared patients with or without optical involvement observing lower plasma levels of IL-6 in those with optic nerve ischemia [65]. These results, however, have not been confirmed by subsequent analyses [66]. Blood levels of PTX-3 and VEGF were elevated in patients with recent optic ischemia [39], and endothelin-1 concentrations were higher in patients with ischemic complications [67]. These observations highlight the fact that the mechanisms of arterial remodelling remain to be defined and the relationship between the arterial remodelling and systemic inflammation is far from obvious. PTX-3 is a locally produced acute phase protein, synthesised by endothelial cells, smooth muscle cells and leukocytes, and plasma PTX may reflect vascular inflammation [68•]. Therefore, PTX-3 levels might better reflect local events than the liver-generated inflammatory biomarkers. In line with this view, relatively low PTX-3 concentrations have been detected in sera from patients with rheumatoid arthritis, PMR and ANCA-associated vasculitides (AAV) confined to isolated involvement of the ear-nose-throat district or orbital granulomas and without vascular involvement [69, 70].

Another unmet need is the identification of response biomarkers able to predict those patients likely to be refractory to first-line therapy and target them for initiation with combination of DMARDs. Such biomarkers would represent an important step toward a personalised therapeutic approach. Osteopontin (OPN) is a glycoprotein participating in Th1 and Th17 differentiation, tissue remodelling and inflammation. A preliminary study has shown that serum OPN at diagnosis might predict subsequent relapse in GCA [71•]. Indeed, OPN concentrations were significantly elevated in patients with active disease compared with those in healthy controls, and baseline OPN values were significantly higher in relapsing versus non-relapsing patients. Considering the pathogenic role of macrophages and angiogenesis in the inflamed arterial wall [12], a recent prospective study analysed the blood levels of multiple soluble molecules associated to macrophage activity or neoangiogenesis, including IL-6, SAA, soluble CD163, calprotectin, chitinase-3-like protein 1 (also known as YKL-40), VEGF, angiopoietin-1 and -2 and Tie2 [72•]. All biomarkers except angiopoietin-1 were elevated in active GCA at baseline. Interestingly, high levels of VEGF and angiopoietin-1 and low levels of YKL-40 predicted rapid achievement of GC-free remission. A selection of biomarkers with a possible prognostic role in TA and GCA is provided in Table 1.

## Serological Biomarkers During Follow-up: Biomarkers of Disease Activity and Relapse Predictors

Disease activity assessment during follow-up remains a difficult task, especially in patients with TA or LV-GCA and arterial remodelling, due to a possible mismatch between the evolution of arterial damage in the face of apparent clinical and laboratory remission [73, 74]. Accordingly, efforts are being made in biomarker research to improve the means for assessing disease activity (Table 1). A predominant challenge has been the identification of an adequate reference standard in order to correlate the value of the candidate biomarker with disease activity. LVV activity is a multi-faceted concept, especially for TA. The available activity criteria, such as NIH criteria or ITAS score, lack sensitivity in the assessment of local inflammation and arterial remodelling [4, 75, 76]. Constitutional symptoms, signs of peripheral ischaemia and increased acute phase reactants may all be considered signs of relapsing TA or LV-GCA. However, these events may occur separately or in combination. Despite acute phase reactants are used to identify recurrent systemic inflammation, ESR and CRP levels during relapses are typically lower than those observed at disease onset [77]. Up to 50% of TA patients believed to be in remission based on absent acute phase response and inflammatory symptoms may have histologic evidence of arterial inflammation or develop arterial remodelling at serial angiographic follow-up [4]. Moreover, a specific limitation of acute phase reactants such as ESR and CRP is the follow-up of patients prescribed IL-6 blockers that normalise CRP levels and may abate inflammatory symptoms associated with disease recurrence (Table 2).

**Table 2:** Linkage of serum biomarkers with the IL-6 axis

Markers depending on IL-6 pathway	ESR	Dependent upon on acute phase reactants synthetised by the liver, including IL-6
	CRP	Mainly produced by the liver under the stimulus of IL-6
	SAA	SAA1 and SAA2 genes are upregulated in the liver by IL-6
Partially independent biomarkers	OPN	Synthesised by activated macrophages and other immune cells. Induced by IL-1β, TNF-α and also IL-6
	YLK-40	Released by monocytes/macrophages and weakly correlates with IL-6
Totally independent biomarkers	PTX-3	Expressed in inflamed arteries and relies on IL-1 and TNF-α
	S100 proteins	Produced at local sites of inflammation and do not correlate with IL-6
	VEGF	Macrophage products which do not correlate with IL-6 concentrations
	angiopoietins	

In patients with GCA, identifying a flare is easier when relapsing cranial or polymyalgic symptoms occur, especially when associated with abnormal ESR or CRP levels [60, 78]. Acute phase reactants are currently used in clinical practice for guiding treatment and identifying relapses, and most clinicians base their evaluation on both clinical improvement and normalisation of inflammatory markers. CRP generally normalises early after initiation of systemic GC, while normalisation of ESR usually takes at least 3–4 weeks [79].

SAA has been reported to be another potential biomarker of systemic inflammation in the setting of active vasculitis, both for TA [30, 80] and GCA [81]. However, evidence is limited, and no advantage over ESR and CRP has been identified so far, since a formal comparison of the accuracy of these biomarkers is lacking.

### Inflammatory Molecules, Cytokines and Cell Adhesion Molecules

In addition to acute phase reactants included in the routine biochemistry panel, other inflammatory molecules have been evaluated during the follow-up of LVVs, including adhesion molecules, which are likely relevant in leukocyte recruitment to the arterial wall. Results are not always concordant between different studies and require further confirmation in independent analyses. Among inflammatory cytokines, higher levels of serum IL-6 and IL-18 have been reported in active TA patients as compared to inactive subjects [35, 36]. Serum concentrations of IL-6 are significantly more sensitive than ESR for active disease, in either untreated or treated GCA patients [82]. Studies comparing the sensitivity of IL-6 over that of CRP in detecting disease activity are lacking, probably due to the very high correlation of these two biomarkers. However, anti-IL-6 receptor therapies directly attenuate the levels of downstream molecules including CRP. In this specific setting, measuring IL-6 rather than CRP might be rationale, even though IL-6 levels usually rise after the first infusions of IL-6 receptor antagonists [83, 84]. A recent paper showed that persistently high levels of IL-6 despite treatment with tocilizumab may predict future relapses [85].

A preliminary small study on 29 patients with TA observed higher plasma levels of the complement protein iC3b (inactivated C3b) and lower levels of the soluble form of the platelet-endothelium adhesion molecule (PECAM-1, also known as CD31) and of iC5b-9 [86].

Rather than a marker of systemic inflammation, plasma PTX-3 may reflect vascular inflammation [68], as its levels in TA did not reflect NIH activity criteria nor ITAS but were rather associated with imaging findings including arterial wall enhancement at MRI and FDG uptake at PET [38, 87].

Goel at al. [88] evaluated the cytokine profile in 32 patients with TA and found that only interferon (IFN)-γ was associated with disease activity, with higher serum levels in active patients. However, longitudinal profiling of patients revealed that treatment efficacy correlated with lower levels of pro-inflammatory cytokines (IFN-γ, IL-6 and IL-23) and higher levels of anti-inflammatory factors such as IL-10 and transforming growth factor (TGF)-β.

Plasma levels of tumour necrosis factor alpha (TNF-α), IL-6, IL-12p40 and intercellular adhesion molecule (ICAM)-1 are higher during relapses of GCA [89, 90, 91•]. Further research identified increased levels of B cell–attracting chemokine-1/CXC motif ligand 13 (BCA1/CXCL13) and soluble IL-2 receptor α (sIL-2Rα) and lower levels of IFN-γ-induced protein 10/CXC motif chemokine 10 (IP10/CXCL10) in active as compared to inactive GCA [92].

Serum OPN is another biomarker whose levels have been correlated with GCA activity [71]*.* During remission, serum OPN levels were similar to those of healthy controls and did not differ between subjects with GC-driven and tocilizumab-driven remission. Considering that OPN synthesis is partially independent from IL-6, the authors concluded that it might be a promising biomarker to monitor patients on IL-6 antagonists.

### Peripheral Leukocyte Immunophenotype

Considering the importance of cell-mediated immunity, it is not surprising that studies have evaluated the correlation of disease activity and treatment effect on the immunophenotype of circulating lymphocytes. This research has proposed a role for Th1 and Th17 cells, although with relevant differences between TA and GCA. In active TA, increased circulating Th1 and Th17 cells were observed, together with raised levels of IL-2, IFN-γ, IL-17A, monocyte chemotactic protein (MCP)-1 and macrophage inflammatory protein (MIP)-1β [93]. Patients prescribed GC therapy showed lower Th1 cytokines such as IL-2 and IFN-γ but not Th17 cytokines. In GCA, treatment with GC therapy suppressed circulating Th17 but not Th1 cells and this paralleled a reduction in the concentration of cytokines promoting Th17 differentiation, such as IL-1β, IL-6 and IL-23, while levels of the Th1 cells producing IL-12 remained unaffected [94]. The authors concluded that Th17 responses were associated with acute manifestation of GCA while persistent Th1 responses might be responsible for relapses during GC tapering. A third, more recent, study assessed the immune phenotype of peripheral leukocytes (T cells, B cells, natural killer cells, dendritic cells, monocytes and granulocytes), observing that disease relapse was associated in TA and GCA with a higher number of circulating Th1, Th17 and T follicular helper cells. In TA, increased numbers of CD8+ T cells in relapsing patients were additionally observed [95]. Th1, Th17 and T follicular helper cells, but not CD8+ T cells, decreased after treatment with biologic agents in GCA and TA. Further studies are required to confirm the different responses of cellular subsets to GC observed in TA and GCA and to define their relevance for pathogenesis and biomarker identification.

### Autoantibodies and B Cell Responses

The use autoantibodies as markers of LVV activity and relapse remains to be clarified. One study identified high titres of IgM anti-endothelial cell antibodies in active TA [96], although it is not clear if these represent pathogenic factors or consequences of arterial injury. Anticardiolipin antibodies have been correlated with fluctuation in disease activity, being present in 74% of patients with GCA flares and in 2% of patients with clinical remission on appropriate treatment [45]. Another study [50] focused on anti-ferritin antibodies, which were observed with highest prevalence in active untreated GCA/PMR (92%), intermediate prevalence in GCA/PMR flares during follow-up (69%) and with lowest prevalence during clinical remission (18%). Ferritin is released by injured cells and the pathogenic relevance of these promising observations is unclear. Beyond autoantibodies, studies regarding cytokine promoting B cell responses such as B cell survival factors activation factor (BAFF) and A proliferation–inducing ligand (APRIL) gave discordant results in TA and GCA [97–99], and further research is required in this field.

### YKL-40

The YKL-40 protein, also known as chitinase-3-like protein 1 (CHI3L1), is a heparin- and chitin-binding lectin that is released by neutrophils, macrophage and vascular smooth muscle cells upon activation. YKL-40 is involved in inflammation, extracellular tissue remodelling and fibrosis. In addition to PTX-3, YKL-40 is believed to be involved in inflammation and remodelling of the extracellular matrix after tissue injury and potentially represents a biomarker of arterial inflammation and remodelling. Serum levels of YKL-40 and IL-6, IL-8, IFN-γ, MMP-2, MMP-9, PTX-3 and OPN have been studied in 40 TA patients [100•]. Although all these parameters were increased in active versus inactive disease, none of them had adequate discriminatory capacity to distinguish between the two groups. A multivariate logistic regression model including ESR, CRP, IL-6, PTX-3 and MMP-9 improved discriminative accuracy between active and inactive TA according to NIH criteria by adding YKL-40 (sensitivity: 85.1%; specificity: 94.3%). However, a limitation of YLK-40 is represented by in vitro reports that synthesis is sensitive to GCs [101]. In contrast, a recent study found that serum levels of YLK-40 in GCA patients were persistently high despite GC treatment [72]. Interestingly, YKL-40 levels were clearly decreased in treatment-free remission. In the same study, strong expression of YKL-40 in temporal artery biopsies (TABs) was observed, mainly at the intima-media border region. This observation led to the hypothesis that YKL-40 is produced predominantly in fully developed GCA with transmural inflammation, and hence may require a longer duration of therapy to induce remission. It is now important to confirm this hypothesis and to verify whether YKL-40 levels may guide GC tapering and reflect arterial damage such as aneurysms or dissection.

### Calprotectin and S100 Family of Calcium-Binding Proteins

The S100 proteins, a family of calcium-binding proteins with pro-inflammatory properties, are expressed and released at local sites of inflammation [102]. Accordingly, they are proposed to play a role in the pathogenesis of multiple rheumatic conditions, thus representing appealing biomarkers of disease activity and course.

The two best-known members of this family are S100A8 and S100A9, which form non-covalent heterodimers, known as calprotectin. This complex, which can act as a damage-associated molecular pattern, is released by monocytes and neutrophils during transendothelial migration [103]. The calprotectin complex is abundant in arteritic lesions and its plasma levels were shown to reflect activity in GCA: despite not being able to outperform traditional biomarkers, the addition of calprotectin and S100A12 levels to a predictive model composed of ESR and CRP improved the ability of the Birmingham Activity Score (BVAS) to discriminate between active and inactive TA and GCA [104•]. However, discordance exists concerning the results of studies in TA. One study found that ESR, CRP, calprotectin and S100A12 levels were similar in patients with active and inactive disease [104•], while another observed higher calprotectin levels in active patients and in those that experienced progressive arterial remodelling during follow-up [105]. Importantly, calprotectin levels did not correlate with IL-6 and the acute phase response [72], thus deserving further research as a marker of arterial inflammation or remodelling, particularly in patients on IL-6 pathway inhibitors.

### Angiogenic Factors

Vasa vasorum neoangiogenesis represents a crucial step in LVVs [12, 13]. VEGF serum levels have been shown to reflect disease activity and arterial inflammation at imaging in GCA [87] but not in TA [106]. There is evidence suggesting that Ang-1/Ang-2 might be involved in GCA, as higher levels of Ang-1 have been reported in active GCA, while increased Ang-2 levels during remission have been observed in patients with imminent disease relapse [72]. Neoangiogenesis requires a tight modulation of pro- and anti-angiogenic factors which include, among others, full-length chromogranin A (CgA) and its fragment vasostatin-1. Interestingly, the anti-angiogenic potential of these two CgA peptides was found to be reduced in TA patients experiencing progressive arterial remodelling [107].

### Matrix Metalloproteinases and Matrix-Remodelling Molecules

MMPs are proteases that degrade components of the extracellular matrix, thus playing a role in several key biological processes including leukocyte extravasation, cell migration and extracellular matrix turnover and remodelling. MMP-9 participates in migration of smooth muscle cells (SMCs) from media to intima, a process leading to intimal hyperplasia [108]. Higher blood levels of MMP-2 and MMP-9 were reported in active vs inactive TA [100], while increased levels of the MMP inhibitor TIMP-1 were observed in active versus inactive GCA [92].

### Circulating Endothelial Precursors

Circulating endothelial precursor cells are markers of endothelial cell damage and repair/angiogenesis. A small number of studies address the number of endothelial precursor cells in the peripheral blood in TA, using different gating strategies to identify these cells (CD309-VEGFR2/CD133/CD34 [106, 109] or CD146/CD31/CD34+, CD45− cells [110]). Their number in peripheral blood has been reported to correlate with disease activity in TA [109, 110], although a more recent study did not confirm these results [106] and the exact mechanism underlying this occurrence is scarcely understood.

## Conclusions

Understanding the significance of blood biomarkers in patients suffering from LVVs would have a variety of potential benefits for clinicians and patients, including reduced diagnostic delay and improved precision medicine by early prediction of responders and refractory subjects and by accurate assessment of disease activity. Currently, the use of biomarkers in LVVs in clinical practice is limited because of their limited variety and availability, high cost, inconsistency between results of different studies and their overall poor discriminative value in distinguishing patients with active and inactive disease, as well those with a benign versus a relapsing course. The widely available ESR and CRP are used in clinical practice, but they do not adequately reflect all disease processes, including clinically silent vascular remodelling. Future studies should stem from improved pathophysiologic knowledge of these conditions and must be based on a prospective assessment with a precise definition of the benchmark variables used to stratify patients.
